# The analgesic effects of insulin and its disorders in streptozotocin‐induced short‐term diabetes

**DOI:** 10.14814/phy2.16009

**Published:** 2024-04-19

**Authors:** Ali Mohammad Basatinya, Javad Sajedianfard, Saeed Nazifi, Saied Hosseinzadeh

**Affiliations:** ^1^ Department of Basic Sciences, School of Veterinary Medicine Shiraz University Shiraz Iran; ^2^ Department of Clinical Science, School of Veterinary Medicine Shiraz University Shiraz Iran; ^3^ Department of Food Hygiene and Public Health, School of Veterinary Medicine Shiraz University Shiraz Iran

**Keywords:** CNS, diabetes, gene, monoamine, pain

## Abstract

Evidence suggests that insulin resistance plays an important role in developing diabetes complications. The association between insulin resistance and pain perception is less well understood. This study aimed to investigate the effects of peripheral insulin deficiency on pain pathways in the brain. Diabetes was induced in 60 male rats with streptozotocin (STZ). Insulin was injected into the left ventricle of the brain by intracerebroventricular (ICV) injection, then pain was induced by subcutaneous injection of 2.5% formalin. Samples were collected at 4 weeks after STZ injection. Dopamine (DA), serotonin, reactive oxygen species (ROS), and mitochondrial glutathione (mGSH) were measured by ELISA, and gene factors were assessed by RT‐qPCR. In diabetic rats, the levels of DA, serotonin, and mGSH decreased in the nuclei of the thalamus, raphe magnus, and periaqueductal gray, and the levels of ROS increased. In addition, the levels of expression of the neuron‐specific enolase and receptor for advanced glycation end genes increased, but the expression of glial fibrillary acidic protein expression was reduced. These results support the findings that insulin has an analgesic effect in non‐diabetic rats, as demonstrated by the formalin test. ICV injection of insulin reduces pain sensation, but this was not observed in diabetic rats, which may be due to cell damage ameliorated by insulin.

## Graphical abstract

The analgesic effects of insulin and its disorders in STZ‐induced short‐term diabetes demonstrated that diabetes (absence of peripheral insulin) increases pain sensation. The results of the formalin test of diabetic groups prove this issue. Insulin injection in healthy groups reduces pain, but this effect is not seen in diabetic groups. According to the supplementary investigations, cellular disorders caused by the complications of the lack of insulin in the central nervous system, such as hyperglycemia, do not reduce the pain sensation in diabetic groups.

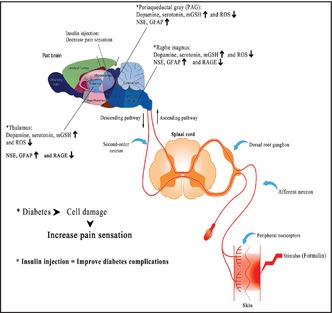

## 1 INTRODUCTION

Pain is a complex experience involving multiple components, including sensory‐discriminative, cognitive‐evaluative, and affective‐emotional processes (Chebbi et al., 2014; Fields, 2005; Millan, 2002), which is defined by the International Association for the Study of Pain (IASP) as pain that occurs as a direct consequence of abnormalities in the somatosensory neural system of known diabetic patients (Akintoye et al., 2020). Pain is the most common symptom of the disease; it is now known that pain is a protective mechanism by which the body responds to noxious stimuli (Świeboda et al., 2013). This effect is lost when the pain becomes a chronic medical condition as seen in patients with diabetic neuropathic pain (DNP), a common complication of diabetes mellitus (DM) (Abbott et al., 2011; Akintoye et al., 2020).

Insulin is well known for its role in glucose and amino acid metabolism but little is known about its function in the central nervous system (CNS) (Kim & Feldman, 2012; van der Heide et al., 2006). The brain has been considered to be an insulin‐independent organ, but basic and clinical research in recent years has shown that this is not true (Blázquez et al., 2014; Duarte et al., 2012). There is evidence that insulin is synthesized in the CNS (Banks, 2004; Woods et al., 2003). In the brain, insulin receptors are expressed by both astrocytes and neurons (Laron, 2009). Neuronal insulin receptors are concentrated at synapses and are components of postsynaptic densities (Chiu et al., 2008; Lee et al., 2005). One of the primary actions of insulin in the CNS is its effect on cognitive function and neurodegenerative disorders (Cunnane et al., 2020; Hölscher, 2020; Mazon et al., 2017).

Based on some findings, it has been hypothesized that insulin resistance may underlie pathological mechanisms leading to central pain (Pappolla et al., 2021). Hyperglycaemia, as one of the major complications of diabetes, alters the sensitivity of experimental animals to various pharmacological agents. Investigators have confirmed that in streptozotocin (STZ)‐induced diabetic rats, the threshold for pain perception is significantly reduced (Ibironke et al., 2004). Insulin exerts its regulatory effects on endoneurial microcirculation, regeneration, and nociception via interaction with its receptor, independent of its hypoglycemic effect (Sugimoto et al., 2008).

Most previous investigations of neuropathic pain have focused on peripheral mechanisms of sensitization but recent concepts agree that central sensitization is also involved in neuropathic pain (Zimmermann, 2001). The periaqueductal gray (PAG) is a midbrain structure involved in the descending control of pain (Millan, 2002; Rahimi et al., 2019). Some glutamatergic neurons project from the PAG to the rostral ventromedial medulla, which suppresses pain signals (Heinricher et al., 2009). Recently, some studies have shown that neuropathic pain can be induced by changes in inhibitory descending pain pathways (Boadas‐Vaello et al., 2016; Monhemius et al., 2001).

Data from magnetic resonance spectroscopy studies suggest that abnormalities occur in the thalamus in DNP. Animal studies using the STZ rat model have provided evidence for the role of the thalamus as a generator or amplifier of diabetic pain (Paulson et al., 2007). Using autoradiography Paulson et al. (2007) demonstrated that rats with STZ‐induced diabetes showed signs of neuropathic pain (Fischer & Waxman, 2010).

The nucleus raphe magnus (NRM) is known to play a critical role in the descending control of nociceptive processing (Fields, 2005; Heinricher & Ingram, 2008). Electrical stimulation produces antinociception and inhibits spinal cord responses (Chebbi et al., 2014; Fields, 2005), and the NRM has been suggested to be a central part of the serotonergic system (Sounvoravong et al., 2004).

Astrocytes are responsible for glucose absorption and metabolism and are therefore the main target during hyperglycemia. Several studies have shown that a change in glial fibrillary acidic protein (GFAP) expression is the main indicator of astrocyte activity. GFAP levels change in the CNS in animal models of diabetes. It is proposed that GFAP has a functional role in the connection of nerve cells and synaptic transmission and that injury stimulates astrocytes to synthesize more GFAP (Afsari et al., 2008; Hashish, 2015; Pang et al., 2017).

Neuron‐specific enolase (NSE) has been identified as an important indicator of nerve tissue damage. NSE is present at low levels in nerve cells and is readily released after tissue damage. Some experimental studies showed that NSE has a neuroprotective role. It has been proposed that NSE is involved in regulating the stress response and regenerative activity after brain damage (Haque et al., 2018). NSE levels are comparatively high in people with diabetes (Anju et al., 2020; Persson et al., 1987; Selakovic et al., 2005).

Advanced glycation end products (AGEs) have been implicated in the pathogenesis of diabetes. AGEs lead to increased secretion of pro‐inflammatory cytokines and oxygen free radicals (reactive oxygen species [ROS]). During hyperglycemia, these substances accumulate in the tissues. AGEs play a key role in the development of diabetic complications. The receptor for advanced glycation end products (RAGE) is responsible for their damaging effects (Al‐Malki, 2013; De Laat et al., 2012; Mercer et al., 2007).

Oxidative stress plays an important role in the pathogenesis of neurodegenerative changes. Tissue damage during hyperglycemia is attributed to oxidative damage caused by increased production of ROS and a decrease in cellular antioxidants such as glutathione (GSH). It is worth noting that there is evidence of a link between hyperglycemia‐induced oxidative stress and the development of neurological damage. Free radicals can spread intracellularly and cause damage to mitochondrial enzymes and DNA, disrupting cell function, and contributing to the pathophysiology of many diseases. The CNS contains a small number of antioxidant enzymes and is therefore potentially susceptible to oxidative damage (Ates et al., 2006, 2007).

In diabetic animals, the synthesis of dopamine (DA) and serotonin and the overall rate of monoamine cycling are altered. It has been found that STZ‐induced diabetic mice are significantly less responsive to the analgesic effect of morphine. It has been shown that monoamine cycling is reduced in the brains of diabetic animals (Takeshita & Yamaguchi, 1998).

In previous studies, intracerebroventricular (ICV) injection of insulin was found to reduce pain sensation in healthy rats, but this effect was not observed in diabetic rats (Dehkordi et al., 2017); therefore, we investigated the pathway of pain transmission and control in the CNS by measuring the level of expression of the NSE, RAGE, and GFAP genes, in addition to measuring the levels of DA, serotonin, ROS, and mitochondrial glutathione (mGSH) in the raphe magnus, thalamus, and PAG nuclei.

## 2 MATERIALS AND METHODS

### 2.1 Laboratory animals

60 adult male Sprague–Dawley rats (60–65 days old) weighing approximately 250–290 g were randomly assigned to different groups. To acclimate to the environment, the animals were kept under the same conditions (12 h dark and 12 h light, temperature (22 ± 2°C), humidity (50%–60%)) for 2 weeks. The animals were divided into six groups (*n* = 10) as follows:

Group 1 (G1)—Non‐diabetic control: 5 μL normal saline, ICV; 50 μL normal saline, SC (subcutaneous injections) in right paw.

Group 2 (G2)—Diabetic control: 5 μL/mU/animal insulin, ICV; 50 μL normal saline, SC in right paw.

Group 3 (G3)—Non‐diabetic with ICV insulin injection: 5 μL/animal insulin, ICV; 50 μL of 2.5% formalin, SC in right paw.

Group 4 (G4)—Non‐diabetic with ICV normal saline injection: 5 μL normal saline, ICV; 50 μL of 2.5% formalin, SC in right paw.

Group 5 (G5)—Diabetics with ICV injection of normal saline: 60 mg/kg STZ, IP; 5 μL normal saline, ICV; 50 μL of 2.5% formalin, SC in right paw.

Group 6 (G6)—Diabetic with ICV insulin injection: 60 mg/kg STZ, IP; 5 μL/mU/animal insulin, ICV; 50 μL of 2.5% formalin, SC in right paw.

### 2.2 Diabetes induction

Prior to the induction of DM in the rats of the diabetic groups, the blood glucose levels of the animals were measured. A single dose of STZ (Sigma‐Aldrich, Cat. No.: S0130‐1G) was administered intraperitoneally to rats in groups 2, 5, and 6 at a dose of 60 mg/kg. STZ was dissolved in cold sodium citrate buffer (Merck, Cat. No.: 106448) at pH = 4.5; before the induction of diabetes, food was withdrawn from the animals' reach for 12 h; then, for 24 h after STZ injection, the animals received 5% dextrose (Merck, Cat. No.: 108346).

### 2.3 Stereotaxic surgery

After 72 h, blood was collected from the tails, and their glucose levels were measured using a glucometer (ACCU—CHECK, Performa model). Rats with plasma glucose levels greater than 250 mg/dL were considered diabetic mice. After the induction of diabetes, the animals were kept for 2 days, weighed, and anesthetized by intraperitoneal injection of the ketamine 80 mg/kg (Alfasan Ketamine 10% 50 mL) and xylazine 8 mg/kg (Alfasan Xylazine 2% 30 mL). The anesthetized animals were placed in the stereotaxic apparatus (Stoelting, Wood Dale, IL, USA), and the cannula implantation sites were based on the coordinates mentioned in the Paxinosis Atlas (AP = −0.8 mm from bregma, L = +1.5 mm from the midline and DV = −3.6 mm from the surface of the skull) (Paxinos et al., 1980; Takeshita & Yamaguchi, 1998). A 22‐gauge needle head was used to make the cannula, and a 29‐gauge dental needle (0.5 mm of the dental needle remains outside the cannula) was used to inject insulin into the left ventricle of the brain.

After the surgery, the animal was given 14 days to recover, and then normal saline and insulin were injected at specified doses in the different groups.

### 2.4 Formalin test

To produce experimental tonic pain in rats, 50 μL of 2.5% formalin (prepared by diluting 38% formalin (Merck, Cat. No.: 818708) with distilled water) was injected subcutaneously (SC) into both the hind paw and the right paw using a 1 mL (27‐gauge) syringe.

The formalin pain test was performed first, followed by the euthanasia of the animals with CO_2_ gas. The raphe magnus nucleus, thalamus, and PAG nucleus were isolated. The samples were then immediately transferred to the freezer where they were kept at −70°C for the next steps (RT‐qPCR and ELISA).

### 2.5 RT‐qPCR assay for GFAP, NSE, and RAGEs

The method was used to assess the expression of GFAP, NSE, and RAGEs mRNA. Brain tissue was isolated and stored at −70°C for RNA extraction. RNX‐Plus solution for total RNA isolation (Sinaclon, Iran, Cat. No.: EX6101) was used to extract of RNA from each sample. The concentration of the extracted RNA was determined using NanoDrop1 C instrument (Thermo Scientific Co., USA). The amount of RNA in the samples was normalized with DEPC water. The Easy™ cDNA Synthesis Kit (Para Tus Inc., Iran, Cat. No.: A101161) was used for cDNA synthesis. Genes expression in each sample was quantified by RT‐qPCR. 16S rRNA was used as the reference internal control gene. The RT‐qPCR mixture contained 12.5 mL SYBR® green real‐time PCR master mix (Para Tus Inc., Iran, Cat. No.: C101022), 0.8 mL cDNA, 9.7 mL DNase‐free water, and 1 mL for each forward and reverse primer. The RT‐qPCR reaction was performed using a Light Cycler device (Roche, Germany). The RT‐qPCR conditions were as follows: 94°C for 5 min as initial denaturation followed by 40 cycles of 95°C for 30 s, 56°C for 30 s, and 72°C for 30 s.

### 2.6 Assay for DA, ROS, serotonin, and mGSH

To assess the mitochondrial function, TCA cycle activity was also evaluated in all samples by measuring the levels of ROS and mGSH factors. The levels of DA and serotonin in the nucleus of the raphe magnus, PAG, and thalamus were measured by ELISA method, given the importance of monoamines, and the effects of insulin on these substances in the mechanism of pain.

DA was measured using a rat sandwich ELISA kit (CUSABIO Company, China). Sensitivity: 0.039 ng/mL, DA Intra‐Assay Precision (Precision within an assay): CV% <8%, Inter‐assay Precision (Precision between assays): CV% <10%, Cod number: CSB‐E08660r.

ROS was measured using a rat sandwich ELISA kit (Sun Long Biotech Co. Ltd, China). Sensitivity: 6 pg/mL, ROS Intra‐Assay Precision (Precision within an assay): CV% <8%, Inter‐assay Precision (Precision between assays): CV% <10%, Cod number: SL1189Ra.

Serotonin was measured using a rat sandwich ELISA kit (CUSABIO Company, China). Sensitivity: 0.4 ng/mL, ROS Intra‐Assay Precision (Precision within an assay): CV% <15%, Inter‐assay Precision (Precision between assays): CV% <15%, Cod number: CSB‐E08364r.

mGSH was measured using a sandwich ELISA kit (Zellbio Company, Germany). Sensitivity: 0.26 mg GSH/L, GSH Intra‐Assay Precision (Precision within an assay): CV% <8%, Inter‐assay Precision (Precision between assays): CV% <10%.

### 2.7 Statistical analysis

All the experiments were performed in triplicate. Results are expressed as mean ± SD. Differences at *p* < 0.05 were considered statistically significant. Statistical analysis of the pain test and ELISA results was performed using one‐way ANOVA followed by Tukey post hoc test. RT‐qPCR data were analyzed using the Roche LightCycler® 96.

## 3 RESULTS

### 3.1 Formalin test

**FIGURE 1:**
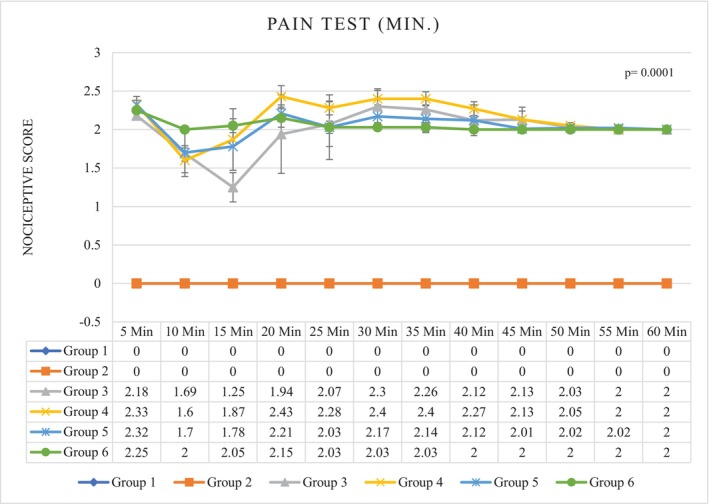
Pain test results after ICV injection (insulin and normal saline). G1. Non‐diabetic control: Normal saline, 5 μL (ICV), normal saline, 50 μL in hind and right paw (SC). G2. Diabetic control: Insulin, 5 μL, 5 mU/animal (ICV), normal saline, 50 μL in hind and right paw (SC). G3. Non‐diabetic with ICV insulin injection: Insulin, 5 μL, 5 mU/animal (ICV), formalin (2.5%), 50 μL in hind and right paw (SC). G4. Non‐diabetic with normal saline ICV injection: Normal saline, 5 μL (ICV), formalin (2.5%), 50 μL in hind and right paw (SC). G5. Diabetes with normal saline ICV injection: Normal saline, 5 μL (ICV), formalin (2.5%), 50 μL in hind and right paw (SC). G6. Diabetes with ICV injection of insulin: Insulin, 5 μL, 5 mU/animal (ICV), formalin (2.5%), 50 μL in hind and right paw (SC). ICV, intracerebroventricular injection; SC, subcutaneous injection. *p* = 0.0001.

After the injection of normal saline in groups 1 and 2 (control), the mean ± SD of the nociceptive score was 0.00 ± 0.00. Pain perception in the first phase (0–5 min) was lower in group 3 (2.18 ± 0.03) than in the other treatment groups. During the interphase (6–15 min), the degree of pain sensation decreased in all treatment groups; the highest and lowest degrees of pain reduction were observed in group 3 (1.47 ± 0.09) and group 6 (2.02 ± 0.05), respectively. During the second phase (16–60 min), pain sensation increased in groups 3, 5, and 6, but the mean pain sensation in group 6 (2.02 ± 0.02) was lower than in the other treatment groups (*p* = 0.0001) (Figure 1; Table 1).

**TABLE 1:** Pain test results after ICV injection (insulin and normal saline).

Groups	Phase		
	First phase	Interphase	Second phase
G1	0.00 ± 0.00^a^	0.00 ± 0.00^a^	0.00 ± 0.00^a^
G2	0.00 ± 0.01^a^	0.00 ± 0.01^a^	0.00 ± 0.00^a^
G3	2.18 ± 0.03^b^	1.47 ± 0.09^b^	2.14 ± 0.07^d^
G4	2.33 ± 0.10^d^	1.73 ± 0.23^c^	2.21 ± 0.03^e^
G5	2.32 ± 0.06^d^	1.73 ± 0.27^c^	2.07 ± 0.08^c^
G6	2.25 ± 0.04^c^	2.02 ± 0.05^d^	2.02 ± 0.02^b^

*Note*: Different letters in a column are used to indicate a significant difference between groups (*p* = 0.0001). G1. Non‐diabetic control: Normal saline, 5 μL (ICV), normal saline, 50 μL in hind and right paw (SC). G2. Diabetic control: Insulin, 5 μL, 5 mU/animal (ICV), normal saline, 50 μL in hind and right paw (SC). G3. Non‐diabetic with ICV insulin injection: Insulin, 5 μL, 5 mU/animal (ICV), formalin (2.5%), 50 μL in hind and right paw (SC). G4. Non‐diabetic with normal saline ICV injection: Normal saline, 5 μL (ICV), formalin (2.5%), 50 μL in hind and right paw (SC). G5. Diabetes with normal saline ICV injection: Normal saline, 5 μL (ICV), formalin (2.5%), 50 μL in hind and right paw (SC). G6. Diabetes with ICV injection of insulin: Insulin, 5 μL,5 mU/animal (ICV), formalin (2.5%), 50 μL in hind and right paw (SC).

Abbreviation: ICV, intracerebroventricular injection; SC, subcutaneous injection.

### 3.2 DA, serotonin, ROS, and mGSH

#### 3.2.1 Thalamus

**FIGURE 2:**
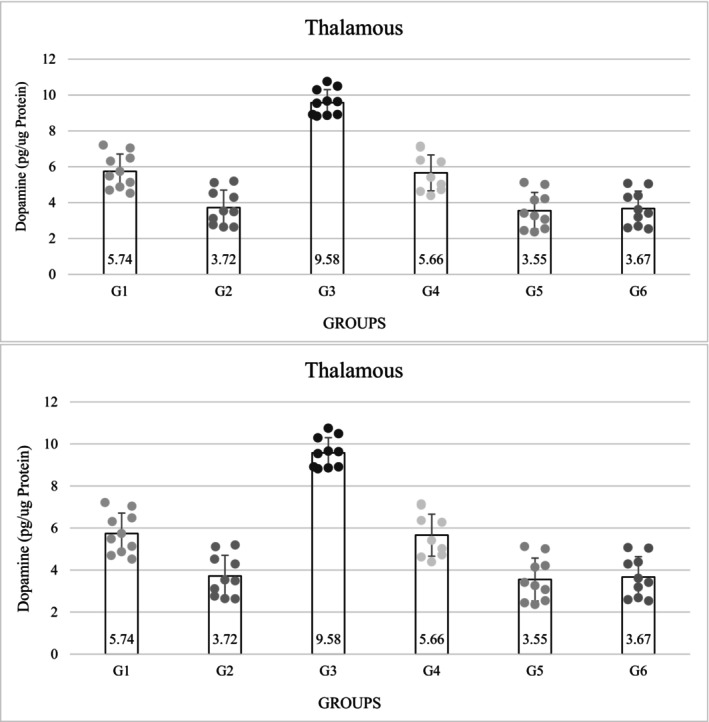
Results of evaluation of dopamine and serotonin level in thalamus (pg/μg protein). G1. Non‐diabetic control: Normal saline, 5 μL (ICV), normal saline, 50 μL in hind and right paw (SC). G2. Diabetic control: Insulin, 5 μL, 5 mU/animal (ICV), normal saline, 50 μL in hind and right paw (SC). G3. Non‐diabetic with ICV insulin injection: Insulin, 5 μL, 5 mU/animal (ICV), formalin (2.5%), 50 μL in hind and right paw (SC). G4. Non‐diabetic with normal saline ICV injection: Normal saline, 5 μL (ICV), formalin (2.5%), 50 μL in hind and right paw (SC). G5. Diabetes with normal saline ICV injection: Normal saline, 5 μL (ICV), formalin (2.5%), 50 μL in hind and right paw (SC). G6. Diabetes with ICV injection of insulin: Insulin, 5 μL, 5 mU/animal (ICV), formalin (2.5%), 50 μL in hind and right paw (SC). ICV, intracerebroventricular injection; SC, subcutaneous injection. *p* = 0.0001.

The concentration of DA in the thalamic nucleus was lower in the diabetic groups than in the non‐diabetic groups. The level of DA in group 5 was decreased (3.55 ± 1.02) (*p* = 0.0001), but in the third group, the level of DA in the thalamus was significantly increased (9.58 ± 0.72), this concentration was significantly higher than in the control groups (*p* = 0.0001). Among the control groups, the measured DA concentration was significantly lower in the diabetic control group than in the non‐diabetic control group (*p* = 0.0001) (Figure 2).

The measured serotonin concentrations were also significantly lower in the diabetic groups than in the non‐diabetic groups (*p* = 0.0001). The serotonin level decreased in group 5 (9.17 ± 1.21), but the third group had the highest serotonin level (17.17 ± 1.04), which was also significant (*p* = 0.0001). The level of serotonin in group 3 was higher than in the control group. The results showed that the level of serotonin was significantly lower in the diabetic control group than in the non‐diabetic control group (*p* = 0.0001). Among the non‐diabetic groups, the third group had the highest amount of serotonin, and this difference was significant (*p* = 0.0001) (Figure 2).

The concentration of oxygen free radicals in the diabetic groups was significantly higher than in the non‐diabetic groups (1, 3 and 4) (*p* = 0.0001). Among the non‐diabetic groups, group 3 (3.68 ± 0.28) had the lowest concentration and among the diabetic groups, group 6 (5.74 ± 0.33) had the highest concentration, which was a significant difference (*p* = 0.0001) (Table 2). The concentration of mGSH in the thalamus decreased significantly in the diabetic groups compared with the non‐diabetic groups (*p* = 0.0001). Among the diabetic groups, group 6 had the highest decrease (163.90 ± 13.50), but among the non‐diabetic groups, group 3 had the highest concentration and was significantly different from group 6. The amount of mGSH in the diabetic control group (175.90 ± 12.28) was significantly lower than in the non‐diabetic control group (*p* = 0.0001) (Table 2).

**TABLE 2:** Results of evaluation of reactive oxygen species (ROS) and mitochondrial glutathione (mGSH) level in thalamus (nmol/g).

Groups	Sample	
	Thalamus	
	ROS	mGSH
G1	4.07 ± 0.03^b^	282.80 ± 8.50^c^
G2	5.50 ± 0.38^c^	175.90 ± 12.28^b^
G3	3.68 ± 0.28^a^	337.90 ± 14.04^c^
G4	4.16 ± 0.34^b^	277.00 ± 8.27^c^
G5	5.72 ± 0.42^c^	165.90 ± 12.28^ab^
G6	5.74 ± 0.33^c^	163.90 ± 13.50^a^

*Note*: Different letters in a column are used to indicate a significant difference between groups (*p* = 0.0001). G1. Non‐diabetic control: Normal saline, 5 μL (ICV), normal saline, 50 μL in hind and right paw (SC). G2. Diabetic control: Insulin, 5 μL, 5 mU/animal (ICV), normal saline, 50 μL in hind and right paw (SC). G3. Non‐diabetic with ICV insulin injection: Insulin, 5 μL, 5 mU/animal (ICV), formalin (2.5%), 50 μL in hind and right paw (SC). G4. Non‐diabetic with normal saline ICV injection: Normal saline, 5 μL (ICV), formalin (2.5%), 50 μL in hind and right paw (SC). G5. Diabetes with normal saline ICV injection: Normal saline, 5 μL (ICV), formalin (2.5%), 50 μL in hind and right paw (SC). G6. Diabetes with ICV injection of insulin: Insulin, 5 μL, 5 mU/animal (ICV), formalin (2.5%), 50 μL in hind and right paw (SC).

Abbreviations: ICV, intracerebroventricular injection; SC, subcutaneous injection.

#### 3.2.2 Nucleus raphe magnus

**FIGURE 3:**
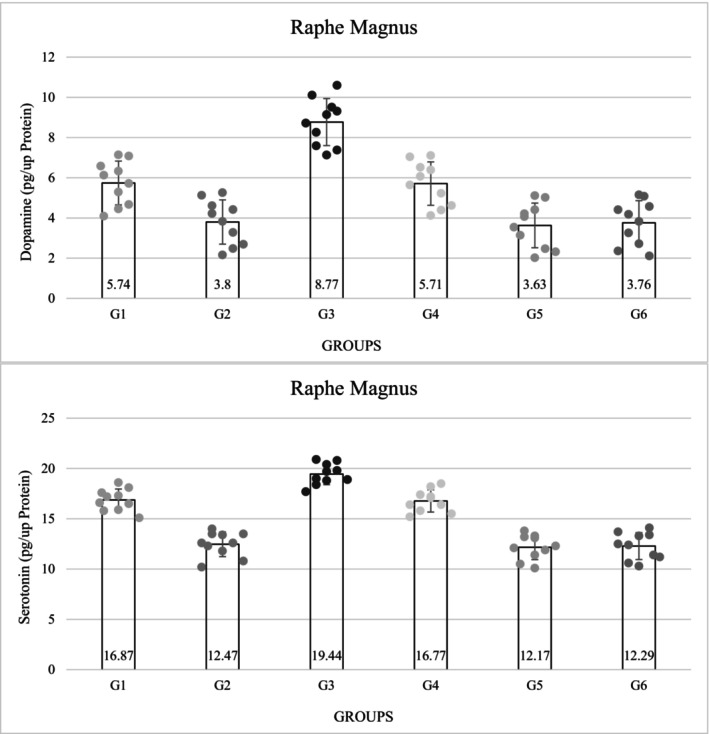
Results of evaluation of dopamine and serotonin level in raphe magnus (pg/μg protein). G1. Non‐diabetic control: Normal saline, 5 μL (ICV), normal saline, 50 μL in hind and right paw (SC). G2. Diabetic control: Insulin, 5 μL, 5 mU/animal (ICV), normal saline, 50 μL in hind and right paw (SC). G3. Non‐diabetic with ICV insulin injection: Insulin, 5 μL, 5 mU/animal (ICV), formalin (2.5%), 50 μL in hind and right paw (SC). G4. Non‐diabetic with normal saline ICV injection: Normal saline, 5 μL (ICV), formalin (2.5%), 50 μL in hind and right paw (SC). G5. Diabetes with normal saline ICV injection: Normal saline, 5 μL (ICV), formalin (2.5%), 50 μL in hind and right paw (SC). G6. Diabetes with ICV injection of insulin: Insulin, 5 μL, 5 mU/animal (ICV), formalin (2.5%), 50 μL in hind and right paw (SC). ICV, intracerebroventricular injection; SC, subcutaneous injection. *p* = 0.0001.

The concentration of DA in the raphe magnus nucleus was significantly lower in the diabetic groups than in the non‐diabetic groups (*p* = 0.0001). The concentration of DA decreased in group 5 (3.63 ± 1.11), but in the third group, which referred to the non‐diabetic group, the concentration of DA was significantly higher than in the other groups (8.77 ± 1.17) (*p* = 0.0001). Among the control groups, the amount of DA in the diabetic control group decreased compared with the non‐diabetic control group and showed a significant difference (*p* = 0.0001) (Figure 3).

The concentration of serotonin was significantly lower in the diabetic groups than in the non‐diabetic groups (*p* = 0.0001). The level of serotonin in group 5 was decreased (12.17 ± 1.23), but in the third group, the level of serotonin was significantly higher than in group 5 (17.64 ± 5.26) (*p* = 0.0001). The amount of serotonin in group 3 was significantly higher than in the control group. The amount of serotonin in the diabetic control group was significantly lower than in the non‐diabetic group 1 (*p* = 0.0001). Among the non‐diabetic groups, the third group had the highest amount of serotonin (Figure 3).

The amount of oxygen free radicals in the diabetic groups was significantly higher than in the non‐diabetic groups (*p* = 0.0001). Group 3 (3.78 ± 0.34) had the lowest concentration and in the diabetic groups, group 5 (5.97 ± 0.31) had the highest amount of oxygen free radicals, the difference was significant (*p* = 0.0001) (Table 3). The concentration of mGSH also showed a significant decrease in the diabetic groups compared with the non‐diabetic groups (*p* = 0.0001). Among the diabetic groups, group 5 (161.30 ± 11.87) showed the greatest decrease, but among the non‐diabetic groups, group 3 showed the highest concentration of mGSH, which was significantly higher than group 6 (*p* = 0.0001). The concentration of mGSH in the diabetic control group (171.40 ± 11.86) was significantly lower than in the non‐diabetic control group (*p* = 0.0001) (Table 3).

**TABLE 3:** Results of evaluation of reactive oxygen species (ROS) and mitochondrial glutathione (mGSH) level in raphe magnus (nmol/g).

Groups	Sample	
	Raphe magnus	
	ROS	mGSH
G1	4.24 ± 0.32^b^	300.10 ± 9.96^b^
G2	5.75 ± 0.31^c^	171.40 ± 11.86^a^
G3	3.78 ± 0.34^a^	369.10 ± 15.90^c^
G4	4.34 ± 0.32^b^	293.50 ± 9.15^b^
G5	5.97 ± 0.31^c^	161.30 ± 11.87^a^
G6	5.94 ± 0.36^c^	161.4 ± 11.40^a^

*Note*: Different letters in a column are used to indicate a significant difference between groups (*p* = 0.0001). G1. Non‐diabetic control: Normal saline, 5 μL (ICV), normal saline, 50 μL in hind and right paw (SC). G2. Diabetic control: Insulin, 5 μL, 5 mU/animal (ICV), normal saline, 50 μL in hind and right paw (SC). G3. Non‐diabetic with ICV insulin injection: Insulin, 5 μL, 5 mU/animal (ICV), formalin (2.5%), 50 μL in hind and right paw (SC). G4. Non‐diabetic with normal saline ICV injection: Normal saline, 5 μL (ICV), formalin (2.5%), 50 μL in hind and right paw (SC). G5. Diabetes with normal saline ICV injection: Normal saline, 5 μL (ICV), formalin (2.5%), 50 μL in hind and right paw (SC). G6. Diabetes with ICV injection of insulin: Insulin, 5 μL, 5 mU/animal (ICV), formalin (2.5%), 50 μL in hind and right paw (SC).

Abbreviations: ICV, intracerebroventricular injection; SC, subcutaneous injection.

#### 3.2.3 PAG

**FIGURE 4:**
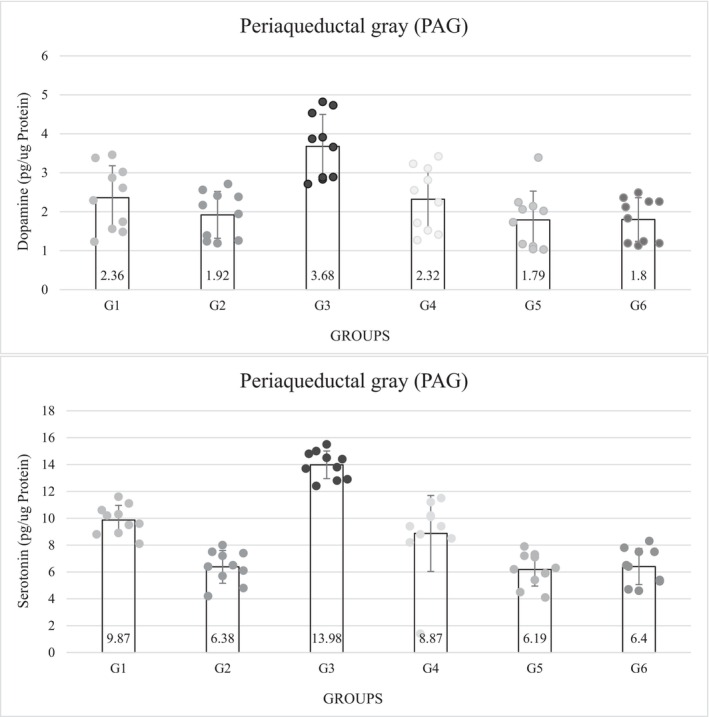
Results of evaluation of dopamine and serotonin level in periaqueductal gray (pg/μg protein). G1. Non‐diabetic control: Normal saline, 5 μL (ICV), normal saline, 50 μL in hind and right paw (SC). G2. Diabetic control: Insulin, 5 μL, 5 mU/animal (ICV), normal saline, 50 μL in hind and right paw (SC). G3. Non‐diabetic with ICV insulin injection: Insulin, 5 μL, 5 mU/animal (ICV), formalin (2.5%), 50 μL in hind and right paw (SC). G4. Non‐diabetic with normal saline ICV injection: Normal saline, 5 μL (ICV), formalin (2.5%), 50 μL in hind and right paw (SC). G5. Diabetes with normal saline ICV injection: Normal saline, 5 μL (ICV), formalin (2.5%), 50 μL in hind and right paw (SC). G6. Diabetes with ICV injection of insulin: Insulin, 5 μL, 5 mU/animal (ICV), formalin (2.5%), 50 μL in hind and right paw (SC). ICV, intracerebroventricular injection; SC, subcutaneous injection. *p* = 0.0001.

The concentration of DA in the PAG nucleus was lower in the diabetic groups was lower than those in the non‐diabetic groups. Among all the experimental groups, the concentration of DA was the lowest in group 5 (1.79 ± 0.74), but in the third group, which was non‐diabetic and received ICV insulin, the amount of PAG DA showed the highest concentration (3.68 ± 0.82); this concentration showed a significant level with other groups (*p* = 0.0001) (Figure 4).

Serotonin concentrations were significantly lower in the diabetic groups than in the non‐diabetic groups (*p* = 0.0001). The serotonin concentration was lowest in group 5 (6.19 ± 1.24) and highest in group 3 (13.98 ± 1.03), which was significant (*p* = 0.0001). The level of serotonin in group 3 was much higher than in the control group. The results of the control groups showed that the concentration of DA was significantly lower in the diabetic control group than in the non‐diabetic control group. Among the non‐diabetic groups, the third group that received ICV insulin had the highest concentration of serotonin and there was a significant difference from all other groups (*p* = 0.0001) (Figure 4).

The concentration of oxygen free radicals was significantly higher in the diabetic groups than in the non‐diabetic groups. Among the non‐diabetic groups, group 3 (4.50 ± 0.27) had the lowest amount of ROS, and among the diabetic groups, group 6 had the highest amount (6.74 ± 0.33), which was a significant difference (*p* = 0.0001). Meanwhile, the difference between the control groups was also significant (*p* = 0.0001) (Table 4). The amount of mGSH in the PAG showed a significant decrease in the diabetic groups compared to the non‐diabetic groups. Among the diabetic groups, groups 5 (165.50 ± 14.85) and 6 (165.50 ± 14.96) showed the greatest reduction; but among the non‐diabetic groups, group 4 had the highest concentration and was significantly different from that of the other diabetic groups. The concentration of mGSH in the diabetic control group (175.90 ± 14.82) was significantly lower than that in the non‐diabetic control group (*p* = 0.0001) (Table 4).

**TABLE 4:** Results of evaluation of reactive oxygen species (ROS) and mitochondrial glutathione (mGSH) level in periaqueductal gray (PAG) (nmol/g).

Groups	Sample	
	PAG	
	ROS	mGSH
G1	5.04 ± 0.03^b^	279.40 ± 19.96^b^
G2	6.54 ± 0.03^c^	175.90 ± 14.82^a^
G3	4.50 ± 0.27^a^	347.70 ± 13.13^c^
G4	5.15 ± 0.32^b^	273.60 ± 19.77^b^
G5	6.50 ± 0.69^c^	165.50 ± 14.85^a^
G6	6.74 ± 0.33^c^	165.50 ± 14.96^a^

*Note*: Different letters in a column are used to indicate a significant difference between groups (*p* = 0.0001). G1. Non‐diabetic control: Normal saline, 5 μL (ICV), normal saline, 50 μL in hind and right paw (SC). G2. Diabetic control: Insulin, 5 μL, 5 mU/animal (ICV), normal saline, 50 μL in hind and right paw (SC). G3. Non‐diabetic with ICV insulin injection: Insulin, 5 μL, 5 mU/animal (ICV), formalin (2.5%), 50 μL in hind and right paw (SC). G4. Non‐diabetic with normal saline ICV injection: Normal saline, 5 μL (ICV), formalin (2.5%), 50 μL in hind and right paw (SC). G5. Diabetes with normal saline ICV injection: Normal saline, 5 μL (ICV), formalin (2.5%), 50 μL in hind and right paw (SC). G6. Diabetes with ICV injection of insulin: Insulin, 5 μL, 5 mU/animal (ICV), formalin (2.5%), 50 μL in hind and right paw (SC).

Abbreviations: ICV, intracerebroventricular injection; SC, subcutaneous injection.

### 3.3 RT‐qPCR

#### 3.3.1 Thalamus

The expression level of GFAP in the diabetic control group (0.30 ± 0.06) (*p* = 0.003) was relatively lower than in the non‐diabetic control group (0.66 ± 0.57), but this difference was not significant. Among the non‐diabetic groups, group 4 (2.58 ± 0.63) showed the highest GFAP expression, which was significantly different from the other groups (*p* = 0.003). In all diabetic groups, the expression level of this gene was lower than in the non‐diabetic groups, and group 6 (0.02 ± 0.02) had the lowest value (Table 5).

**TABLE 5:** Results of evaluation of GFAP, NSE, and RAGE in thalamus.

Groups	Sample		
	Thalamus		
	GFAP	NSE	RAGE
G1	0.66 ± 0.57^a^	0.66 ± 0.57^a^	0.66 ± 0.57^a^
G2	0.3 ± 0.06^a^	0.00 ± 0.01^a^	0.0 ± 0.01^a^
G3	0.86 ± 1.36^a^	1.24 ± 1.13^a^	0.0 ± 0.00^a^
G4	2.58 ± 0.63^b^	0.75 ± 0.65^a^	1.62 ± 2.79^a^
G5	0.07 ± 0.08^a^	2.45 ± 4.11^a^	0.0 ± 0.00^a^
G6	0.02 ± 0.02^a^	0.02 ± 0.01^a^	0.01 ± 0.02^a^

*Note*: Different letters are used to indicate a significant difference between groups. GFAP: *p* = 0.003. NSE: *p* = 0.578. RAGE: *p* = 0.472. G1. Non‐diabetic control: Normal saline, 5 μL (ICV), normal saline, 50 μL in hind and right paw (SC). G2. Diabetic control: Insulin, 5 μL, 5 mU/animal (ICV), normal saline, 50 μL in hind and right paw (SC). G3. Non‐diabetic with ICV insulin injection: Insulin, 5 μL, 5 mU/animal (ICV), formalin (2.5%), 50 μL in hind and right paw (SC). G4. Non‐diabetic with normal saline ICV injection: Normal saline, 5 μL (ICV), formalin (2.5%), 50 μL in hind and right paw (SC). G5. Diabetes with normal saline ICV injection: Normal saline, 5 μL (ICV), formalin (2.5%), 50 μL in hind and right paw (SC). G6. Diabetes with ICV injection of insulin: Insulin, 5 μL, 5 mU/animal (ICV), formalin (2.5%), 50 μL in hind and right paw (SC).

Abbreviations: GFAP, glial fibrillary acidic protein; ICV, intracerebroventricular injection; NSE, neuron‐specific enolase; RAGE, receptor for advanced glycation end product; SC, subcutaneous injection.

The level of NSE gene expression was relatively lower in the diabetic control group than in the non‐diabetic control group. Among the diabetic groups, the highest expression level was observed in group 5 (2.45 ± 4.11) and the lowest in group 2 (0.00 ± 0.01). Among the non‐diabetic groups, the highest expression level was observed in group 3 (1.24 ± 1.13) and the lowest in group 1 (0.66 ± 0.57) (*p* = 0.578) (Table 5).

The results of RAGE gene expression showed that this gene was not expressed in most of the experimental groups. Among the control groups, group 2 (0.00 ± 0.01) had a lower value than the non‐diabetic control group. Group 4 (1.61 ± 2.79) had the highest level of expression compared to all groups. In the case of diabetic groups, group 6 (0.01 ± 0.02) showed a higher expression level (*p* = 0.472) (Table 5).

#### 3.3.2 Nucleus raphe magnus

The expression level of GFAP was lower in the diabetic control group (0.31 ± 0.18) than in the non‐diabetic control group (0.66 ± 0.57), but this difference was not significant. Among the non‐diabetic groups, the expression level of this gene was relatively lower in group 3 (0.24 ± 0.37) and group 4 (0.31 ± 0.31) compared with group 1. In all diabetic groups, the expression level of this gene was relatively increased; the highest value was seen in group 5 (0.95 ± 1.15) (*p* = 0.624) (Table 6).

**TABLE 6:** Results of evaluation of GFAP, NSE, and RAGE in raphe magnus.

Groups	Sample		
	Raphe magnus		
	GFAP	NSE	RAGE
G1	0.66 ± 0.57^a^	0.66 ± 0.57^a^	0.66 ± 0.57^a^
G2	0.31 ± 0.18^a^	0.0 ± 0.00^a^	0.0 ± 0.00^a^
G3	0.24 ± 0.37^a^	0.25 ± 0.28^a^	0.01 ± 0.02^a^
G4	0.31 ± 0.31^a^	0.61 ± 0.96^a^	0.0 ± 0.00^a^
G5	0.95 ± 1.15^a^	0.03 ± 0.06^a^	0.0 ± 0.00^a^
G6	0.75 ± 0.46^a^	0.0 ± 0.01^a^	0.69 ± 1.20^a^

*Note*: Different letters in a column are used to indicate a significant difference between groups. GFAP: *p* = 0.624. NSE: *p* = 0.344. RAGE: *p* = 0.353. G1. Non‐diabetic control: Normal saline, 5 μL (ICV), normal saline, 50 μL in hind and right paw (SC). G2. Diabetic control: Insulin, 5 μL, 5 mU/animal (ICV), normal saline, 50 μL in hind and right paw (SC). G3. Non‐diabetic with ICV insulin injection: Insulin, 5 μL, 5 mU/animal (ICV), formalin (2.5%), 50 μL in hind and right paw (SC). G4. Non‐diabetic with normal saline ICV injection: Normal saline, 5 μL (ICV), formalin (2.5%), 50 μL in hind and right paw (SC). G5. Diabetes with normal saline ICV injection: Normal saline, 5 μL (ICV), formalin (2.5%), 50 μL in hind and right paw (SC). G6. Diabetes with ICV injection of insulin: Insulin, 5 μL, 5 mU/animal (ICV), formalin (2.5%), 50 μL in hind and right paw (SC).

Abbreviations: GFAP, glial fibrillary acidic protein; ICV, intracerebroventricular injection; NSE, neuron‐specific enolase; RAGE, receptor for advanced glycation end product; SC, subcutaneous injection.

The level of NSE gene expression was lower in all groups compared with the non‐diabetic control group. Among the diabetic groups, the highest level of expression was observed in group 5 (0.03 ± 0.06). Among the non‐diabetic groups, group 4 (0.61 ± 0.96) had the highest expression level compared with group 1 (0.66 ± 0.57) (*p* = 0.344) (Table 6).

The RAGE gene expression level in the diabetic groups was the highest in group 6 (0.69 ± 1.20). The RAGE gene was not expressed in group 2 (diabetic control) (0.00 ± 0.00). Group 6 (0.69 ± 1.20) had the highest expression level among the diabetic groups, even compared with the non‐diabetic control group (Table 6) (*p* = 0.353).

#### 3.3.3 PAG

The expression level of GFAP in the diabetic control group (0.37 ± 0.33) was relatively lower than in the non‐diabetic group (0.66 ± 0.57), but this difference was not significant. Among the non‐diabetic groups, group 4 (2.77 ± 2.51) showed the highest expression of GFAP. In the diabetic groups, 5 (3.65 ± 4.11) and 6 (2.39 ± 2.24), the expression level of this gene was higher than in the control groups, and among them, group 5 (3.65 ± 4.11) had the highest expression value (*p* = 0.312) (Table 7).

**TABLE 7:** Results of evaluation of GFAP, NSE, and RAGE in periaqueductal gray (PAG).

Groups	Sample		
	PAG		
	GFAP	NSE	RAGE
G1	0.66 ± 0.57^a^	0.66 ± 0.57^a^	0.33 ± 0.57^a^
G2	0.37 ± 0.33^a^	0.04 ± 0.07^a^	0.00 ± 0.00^a^
G3	0.14 ± 0.21^a^	0.49 ± 0.75^a^	0.00 ± 0.00^a^
G4	2.77 ± 2.51^a^	0.95 ± 1.65^a^	0.00 ± 0.00^a^
G5	3.65 ± 4.11^a^	0.01 ± 0.02^a^	0.00 ± 0.00^a^
G6	2.39 ± 2.24^a^	0.12 ± 0.10^a^	0.00 ± 0.00^a^

*Note*: Different letters in a column are used to indicate a significant difference between groups. GFAP: *p* = 0.312. NSE: *p* = 0.685. RAGE: *p* = 0.458. G1. Non‐diabetic control: Normal saline, 5 μL (ICV), normal saline, 50 μL in hind and right paw (SC). G2. Diabetic control: Insulin, 5 μL, 5 mU/animal (ICV), normal saline, 50 μL in hind and right paw (SC). G3. Non‐diabetic with ICV insulin injection: Insulin, 5 μL, 5 mU/animal (ICV), formalin (2.5%), 50 μL in hind and right paw (SC). G4. Non‐diabetic with normal saline ICV injection: Normal saline, 5 μL (ICV), formalin (2.5%), 50 μL in hind and right paw (SC). G5. Diabetes with normal saline ICV injection: Normal saline, 5 μL (ICV), formalin (2.5%), 50 μL in hind and right paw (SC). G6. Diabetes with ICV injection of insulin: Insulin, 5 μL, 5 mU/animal (ICV), formalin (2.5%), 50 μL in hind and right paw (SC).

Abbreviations: GFAP, glial fibrillary acidic protein; ICV, intracerebroventricular injection; NSE, neuron‐specific enolase; RAGE, receptor for advanced glycation end product; SC, subcutaneous injection.

The level of NSE gene expression was lower in the diabetic control group than in the non‐diabetic control group. Among the diabetic groups, the highest level of expression was observed in group 6 (0.12 ± 0.01) and the lowest level in group 5 (0.01 ± 0.02). Among the non‐diabetic groups, group 4 (0.95 ± 1.65) had the highest level of expression compared with the non‐diabetic control group (*p* = 0.685) (Table 7).

The expression of the RAGE gene did not occur in any of the groups compared with the non‐diabetic control group (*p* = 0.458) (Table 7).

## 4 DISCUSSION

Recent experimental evidence has suggested that insulin has analgesic effects by acting on dopaminergic, and serotonergic analgesic pathways, among others (Anuradha et al., 2004; Dehkordi et al., 2017; Takeshita & Yamaguchi, 1997). In this study, ICV insulin injection produced an analgesic effect in rats. ICV insulin injection in non‐diabetic rats led to a moderation of nociceptive responses during formalin tests; however, this effect was not seen in diabetic rats. They were able to modulate pain responses for a longer period of time. In the current study, we therefore aimed to explain the mechanisms behind this effect, focusing on cell factors in the thalamus, raphe magnus, and PAG.

Our results showed that the concentration of DA, serotonin, and mGSH decreased in the nuclei of the thalamus, raphe magnus, and PAG, and the level of ROS increased. In addition, the expression levels of the NSE and GFAP genes were increased in the nuclei. These results confirm the findings of the formalin test on the analgesic effect of insulin in non‐diabetic rats.

The role of insulin in the CNS is less well understood. However, there is increasing evidence showing neurotrophic, neuromodulatory, and neuroendocrine activity, as well as synaptic plasticity and effects on neurotransmitter release, particularly monoamines (Gasparini et al., 2002; Gerozissis, 2003; Gispen & Biessels, 2000). In addition to its central role, insulin may also modulate neuronal processes in the CNS (Lázár et al., 2020). Molecular mechanisms might contribute to the pathophysiology of diabetic neuropathy and pain (Misawa et al., 2009). Misawa et al. (2009) has suggested that hyperglycemia may reduce the nodal potassium conductance, thereby potentially affecting axonal excitability. Calcium channels have also been implicated in cellular changes in DM (Fischer & Waxman, 2010).

Diabetes may alter nociceptive thresholds (Zhai et al., 2016). Previous animal studies have shown that nociceptive thresholds are lowered by acute hyperglycemia; this effect has been attributed to the hyperinsulinemia induced by hyperglycemia. Insulin stimulates the Na^+^/K^+^ ATPase, and this could alter nerve function directly through stimulation of the ATPase or indirectly through changes in ion distribution. Another mechanism is that a hyperglycemic state induces an excess of intracellular sorbitol in tissues, which increases intracellular osmotic pressure, modulating several ionic conductances and increasing Ca^2+^ influx and membrane depolarization, which are known to increase pain sensitivity. Forman et al. found that plasma hypothalamic plasma levels of the endogenous opioid peptides were reduced in rats after STZ‐induced diabetes (Ibironke et al., 2004; Viana et al., 2001). Painful diabetic neuropathy impairs motor and sensory conduction velocity and nerve blood flow (Hoybergs & Meert, 2007; Saini et al., 2004). Stress increases the glycation of the Na^+^/K^+^‐ATPase and may play a role in the reduction of motor nerve conduction velocity as often seen in diabetic animal models (Singh et al., 2014).

Hyperglycemia is known to lower the pain threshold and increase pro‐inflammatory cytokines leading to both the development and progression of hyperalgesia and allodynia in rats (Taliyan & Sharma, 2012; Yano et al., 2006). Hyperglycemia has been shown to reduce to decrease the antinociceptive effect of opioids. Several mechanisms have been proposed, including such as activation of NMDA, PKA, PKC; receptor desensitization; increased oxido‐nitrosative stress; cytokines; and NO levels (Chen et al., 2009; Grover et al., 2000; Obrosova et al., 2007; Shukla et al., 2006; Taliyan et al., 2010).

Several mechanisms have been proposed to explain hyperglycemia‐induced deficits in motor and sensory nerve conduction velocities. Diabetes‐induced endothelial dysfunction, resulting in decreased nerve blood flow, and endoneurial hypoxia play a key role in functional and morphological changes in the diabetic nerve (Manschot et al., 2003; Obrosova, 2009).

Decreases in central insulin levels or compensatory changes in cortical insulin neurotrophic receptor expression may contribute to CNS deterioration in insulin‐dependent diabetes (Taliyan et al., 2010). Insulin has been shown to have beneficial effects on several manifestations of diabetic neuropathy, including reduction in formalin test hyperalgesia, improvement in sensory and motor nerve conduction velocity, nerve blood flow, and formalin test hyperalgesia. In vivo, nerve conduction velocity measurements show a significant decrease early after diabetes induction is reversed by insulin therapy (Taliyan & Sharma, 2012). Advanced imaging techniques have shown that insulin resistance leads to microcirculatory dysfunction in the brain, resulting in cerebral hypoperfusion (Pappolla et al., 2021).

Numerous animal studies have suggested a specific role for DA in pain modulation (Martikainen, 2009) in the thalamus and PAG (Jarcho et al., 2012; Li et al., 2016). Serotonin (5‐HT) has been implicated in pain perception and modulation. The analgesic effects of serotonin and the role of the descending serotonergic pathway in pain inhibition are well recognized (Martikainen, 2009). Stimulation of the PAG was found to cause serotonin release in the spinal cord and intrathecal administration of 5‐HT agonists induced antinociception (Ossipov et al., 2010). Consistent with previous research, this study showed a decrease in DA and serotonin levels in all diabetic groups. In the groups receiving ICV insulin, these effects were reversed and DA and serotonin levels increased. In addition, levels of ROS increased in the diabetic groups, and levels of mGSH decreased in these groups; both of these effects changed after ICV insulin injection so that ROS decreased and mGSH increased.

DA has been found to be significantly reduced in diabetic rats, which may be due to a reduction in DA synthesis and turnover in the CNS. In addition, a decrease in brain serotonin levels in diabetes may be caused by a reduction in amino acids with a consequent decrease in 5‐HT synthesis (Al‐Brakati et al., 2020). The levels of DA‐degrading enzymes, including monoamine oxidases (MAO) A and B, are also increased in diabetic mice, and these increased levels result in increased DA clearance. In addition, the experiments show that the altered expression of the MAO A and B is a direct consequence of the loss of insulin signaling in the neurons and glia (Kleinridders et al., 2015). The decrease in brain tryptophan alone may be sufficient to cause a decrease in the rate of 5‐HT synthesis. The reduction in brain tryptophan in diabetic rats appears to be due to an actual decrease in plasma tryptophan concentration, as well as to a large increase in the branched‐chain amino acids (leucine, isoleucine, and valine), which compete with tryptophan for uptake into the brain (Trulson et al., 1986).

Insulin is known to modulate the serotonin system in the CNS. Insulin administration attenuates the neurochemical changes induced by STZ‐treated diabetes, as indicated by increased serotonin levels. Insulin administration causes an increase in the concentration of tryptophan (a serotonin precursor) and serotonin in the rat brain, which is reversed by insulin administration (Gupta et al., 2014). The fact that insulin regulates DA signaling has been established in animal studies (Eisenstein et al., 2015). Insulin regulates the dopaminergic system through at least three molecular mechanisms: (1) insulin modifies DA or regulates the protein expression of the DA‐degrading enzymes MAO and the dopamine transporter (DAT), (2) insulin regulates the uptake of released DA by induction of DAT expression, and (3) it alters the spike frequency of dopaminergic neurons and cholinergic interneurons (Kleinridders & Pothos, 2019).

Oxidative stress is considered to be a major cause of diabetic complications. Hyperglycemia leads to increased glucose oxidation in mitochondria and the release of a large amount of ROS; This disrupts normal cellular function and leads to dysfunction (Al‐Brakati et al., 2020). Diabetic mice show mitochondrial dysfunction in the brain. Loss of insulin signaling in the CNS affects mitochondrial function: (1) reduced mitochondrial activity due to decreased expression of electron transport chain proteins; (2) increased monoamine oxidase levels due to a loss of insulin action to suppress MAO gene expression; and (3) changes in the mitochondrial morphology (Kleinridders et al., 2015). The potential destructive effects of free radicals are controlled by cellular antioxidant defenses such as GSH. The distribution of GSH in diabetic tissues of diabetics may play an important role in the pathology of diabetes. Increasing GSH levels may reduce oxidative stress in diabetes (Hamdy & Taha, 2009).

Neurons and glia can synthesize GSH, and astrocytes can release GSH into the extracellular space where it is metabolized into components that are taken up by neurons and resynthesized into GSH (Hauser & Hastings, 2013). GSH is a major cellular antioxidant. The de novo synthesis of GSH is dependent on the utilization of L‐glutamine, but L‐glutamine levels have been reported to be reduced in patients with type 2 diabetes mellitus (T2DM) (Calabrese et al., 2012; Cruzat et al., 2014; Menge et al., 2010; Newsholme et al., 2016). Depletion of antioxidants such as GSH increases the susceptibility of the CNS to oxidative changes in DM and untreated DM results in lower GSH levels in several brain regions (Ates et al., 2007). Short‐term diabetes can induce GSH depletion, increased ROS levels, and apoptosis as early as 4 days after STZ administration (Ghosh et al., 2005). Reduction in mGSH has been implicated in many diseases such as DM (Dwivedi et al., 2020).

Insulin has been shown to have antioxidant properties. The generation of ROS by mitochondrial oxidative phosphorylation is attenuated by insulin through the regulation of the expression of uncoupling proteins. In addition, the expression of NADPH oxidases is inhibited by insulin. Insulin may promote the scavenging of ROS through the production of intracellular antioxidants, and the upregulation of various antioxidant enzymes (Newsholme et al., 2007; Rochette et al., 2014). Full electron transport chain mitochondrial respiration is significantly reduced by 30%–40% in STZ diabetic rats, which can be improved by insulin (Fernyhough et al., 2010). In cases where oxidative stress causes GSH impairment; such as diabetes, the hyperinsulinemic state improves the intracellular GSH redox state (Bravi et al., 2006).

Non‐diabetic rats treated with insulin tended to show thermal and mechanical hypoalgesia (Sugimoto et al., 2008). It has been found that diabetes not only causes changes in body weight and food intake but also has significant effects on the neuroendocrine system. These effects were completely reversed by insulin treatment (Barber et al., 2003).

In this study, the expression of GFAP and NSE genes in the affected brain nuclei shows that diabetes has increased the expression of these genes. In addition, in some cases, formalin‐induced pain enhanced this effect. This study concluded that insulin injection improved the expression level of the GFAP and NSE genes, but it can be said that insulin injection had different relative effects on gene expression in the brain nuclei due to the degree of disruption caused by diabetes. Acute severe pain also reduces insulin sensitivity, mainly by affecting non‐oxidative glucose metabolism (Greisen et al., 2001).

It has been suggested that NSE is a biomarker for assessing neuronal damage (neuropathy marker); when the nerve is injured, NSE is released into the cerebrospinal fluid (Sirisha et al., 2021). Increased levels of NSE have been observed after cortical brain injury (Tomaszewski, 2015). NSE expression was higher in STZ‐induced diabetic animals, and the number of NSE‐expressing neurons increased in the CNS (Dincel & Yildirim, 2016). In diabetic patients, high glucose levels were associated with higher serum NSE concentrations (Elshorbagy et al., 2019). Previous studies suggest that insulin may inhibit brain injury (Chen et al., 2014). It has been reported that NSE is higher in DM patients when compared to normal controls (Li et al., 2013). In the present study, we observed a significant reduction in NSE after insulin treatment in all treatment groups (non‐diabetic and diabetic) compared with DM groups without treatment. This may be an indication of nerve regeneration, as a reduction in NSE is a marker of improvement in nerve structure and function (Sirisha et al., 2021).

GFAP levels were higher in patients with mass lesions than in those with diffuse brain injury (Tomaszewski, 2015). A significant reduction in GFAP expression in the brain parenchyma was observed in STZ‐induced diabetic animals (Dincel & Yildirim, 2016). The reduced GFAP released by astrocytes and the reduced GFAP area ratio in STZ‐induced diabetic rats may cause changes in the functional properties of the astrocytes and weaken their ability to provide neuronal support in the CNS (Hashish, 2015). A decrease in the GFAP expression in DM is thought to be due to the apoptosis/degeneration of astrocytes (Dincel & Yildirim, 2016). Previous studies have shown that the percentage of GFAP‐immunoreactive astrocytes decreases in the cerebellum of STZ‐induced diabetic rats. This finding is consistent with the fact that GFAP content was also reduced in the olfactory bulb of STZ diabetic animals. There was also a reduction in the number of GFAP astrocytes in the gray matter of the spinal cords of STZ‐induced diabetic rats recognized. This may be due to oxidative damage associated with diabetes. Reduced expression is associated with detrimental conditions in the CNS several of which occur in diabetes. Disruption of the blood–brain barrier, reduced white matter vascularization, and impaired long‐term potentiation (LTP) have been reported in GFAP knock‐out mice. These explanations suggest a link between reductions in GFAP and diabetes‐induced CNS complications (Bouchard et al., 2002; Chehade et al., 2002; Coleman et al., 2010; Kamal et al., 2000; Pekny & Pekna, 2004).

Insulin treatment improves glutamate uptake and prevents the diabetes‐induced increase in sodium‐independent glutamate uptake in glial cells, suggesting that insulin may improve the antioxidant status of the brain in diabetes. This is consistent with our results as insulin treatment caused an increase in the GFAP expression. It has been documented that insulin‐treated rats had increased astrocytic GFAP as compared to diabetic rats (Hashish, 2015); therefore, insulin is of great importance in improving the function of astrocytes, the dysfunction of which is implicated in the CNS complications of diabetes. Another effect of insulin treatment was to modify an increase in glutamate uptake in diabetic rats. In diabetic animals, sodium‐dependent and sodium‐independent glutamate uptake was greater in glial vesicles and insulin can ameliorate these effects (Coleman et al., 2010). Insulin affects astrocyte morphology and GFAP expression in rat astrocyte cultures and is important for astrocyte differentiation and maintenance of astrocyte function (Coleman et al., 2004).

Protein glycation and the formation of AGEs play an important role in diabetic complications, such as neuropathy. AGEs bind to plasma membrane RAGE to alter intracellular signaling, gene expression, and the release of pro‐inflammatory molecules and free radicals, and thus they play an important role in the pathogenesis of diabetic neuropathy. The formation of AGEs by reactive di‐carbonyls has been shown to play a key role in the pathogenesis of sensory neuron damage (El‐Mesallamy et al., 2011; Jack & Wright, 2012; Singh et al., 2014). Long‐term hyperglycemia in type 1 diabetes mellitus (T1DM) leads to increased expression of receptors for RAGE and accelerates the formation of RAGE ligands, including AGEs (Du et al., 2022; Khalid et al., 2022; Le Bagge et al., 2020). Insulin treatment reduces the activation of ROS and AGEs, and the expression levels of RAGE in diabetic rats. Insulin treatment inhibited ROS, AGEs, and RAGE and contributed to the expansion of diabetic encephalopathy (Hawkins et al., 2007; Sun et al., 2015).

## 5 CONCLUSION

The current study showed that ICV insulin injection reduced pain sensation, increased the levels of monoamines, and antioxidant factors, and reduced the expression level of the NSE and GFAP genes. However, this effect was not observed in the diabetic rats. This seems to be due at least in part to hyperglycemia, the destructive effect of diabetes on sensory and motor neurons (such as a decrease in the speed of nerve impulses), causing damage to the cerebrovascular system, and disruption of astrocytes.

Our study suggests that central insulin injection could improve pain sensation and cell damage caused by diabetes.

## AUTHOR CONTRIBUTIONS

Ali Mohammad Basatinya and Javad Sajedianfard collected data, designed the project, and performed the statistical analysis. Saeed Nazifi accomplished ELISA analysis. Saied Hosseinzadeh completed the RT‐qPCR assay. Ali Mohammad Basatinya, Javad Sajedianfard, and Saied Hosseinzadeh reviewed and revised the final version of the manuscript. All authors revised and approved the final submission and agreed to all aspects of this work.

## CONFLICT OF INTEREST STATEMENT

The authors have no relevant financial or non‐financial interests to disclose.

## ETHICS STATEMENT

All experiments approved by State Animal Ethics Committee, Shiraz University, Shiraz, Iran (96GCU1M1293).

## Data Availability

The datasets generated during and/or analyzed during the current study are not publicly available, due to individual privacy and protection of critical information from illegal disclosure, but are available from the corresponding author upon reasonable request.
